# Separated Energy Domains in the Sequential Hydration of a Benzonitrile Radical Cation

**DOI:** 10.1021/prechem.5c00294

**Published:** 2025-12-31

**Authors:** Bingzheng Wu, Shirong Wang, Xin Xu, Sai Duan

**Affiliations:** † State Key Laboratory of Porous Materials for Separation and Conversion, Shanghai Key Laboratory of Molecular Catalysis and Innovative Materials, MOE Key Laboratory of Computational Physical Sciences, Research Center for Chemical Theory, Department of Chemistry, 12478 Fudan University, Shanghai 200433, P. R. China; ‡ Hefei National Laboratory, Hefei 230088, P. R. China

**Keywords:** weak interactions, radical-water clusters, distonic cations, configuration search, spectral simulation, potential energy surface, XYG3-type doubly hybrid

## Abstract

The sequential hydration of organic radical cations provides a model system to investigate the intriguing interaction between the radical and solvent with unconventional carbon-based ionic hydrogen bonds. In this context, a benzonitrile radical cation (BN^•+^) bonded to water (H_2_O) has been experimentally investigated by infrared (IR) spectrum with a single water molecule and mass spectra with up to seven water molecules. In this work, we performed a comprehensive potential energy surface search at the highly accurate doubly hybrid density functional theory (XYGJ-OS) and couple-cluster level to exhaustively explore the low-lying structures of BN^•+^–(H_2_O)_*n*=1–6_. For *n* = 1, an isomer where water binds to the *ortho*-C–H group is determined as the most populated configuration at room temperature due to its low energy and chirality. Calculated thermal-averaged IR spectrum reveals that this ortho-isomer plays an essential role in quantitative reproduction of the experimentally measured counterpart of BN^•+^–H_2_O. As the number of water molecules increases, a kind of distonic cation including a cycle that consists of a protonated water cluster, cyano group, and hydroxyl substituent is identified as the kinetically dominant species at ambient conditions, which requires overcoming an energy barrier as high as 30 kcal/mol to isomerize to the most stable configurations. The characteristic protonated water cluster moiety in the kinetically stable isomers results in strong ionic hydrogen bonds, which are responsible for the absence of signals of BN^•+^–(H_2_O)_*n*≤3_ in experimental mass spectra. Calculated IR spectra demonstrate that the range between 1700 and 2700 cm^–1^ is the diagnostic region for distinguishing kinetically and thermodynamically stable isomers. These findings establish a paradigm for the interaction between BN^•+^ and water, paving the way for a further understanding of the hydration of organic radical cations.

## Graphical abstract

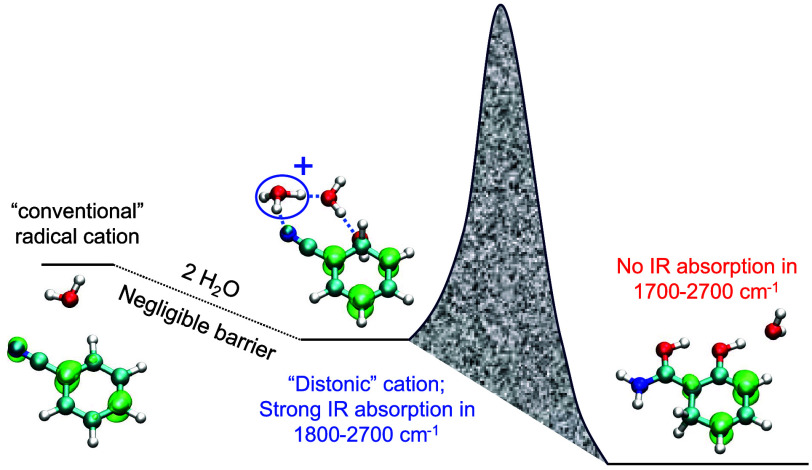

## 1 Introduction

Interactions between radicals and water molecules are ubiquitous in both biological^1^ and astrochemical^2^ processes, providing insights into life processes^3^ and the origins of prebiotic molecules.^4^ It is a consensus that the proton affinity of water clusters increases monotonically with their sizes.^5^ Furthermore, radicals are well-known for their hallmark reactivity, arising from unpaired electrons.^6^ As a result, it is expected that in radical cation/water cluster systems, locations of the charge and the unpaired electron would eventually become spatially separated,^7^ leading to the so-called “distonic ions”.^8^

Owing to the recent detection of benzonitrile (BN) in outer space,^10,11^ exploring the stepwise hydration of its radical cation (BN^•+^) is therefore a valuable model system for understanding not only the functionalized organic solvent but also the interstellar aromatic molecule. To this end, infrared photodissociation (IRPD) spectroscopy and mass spectrometry were used for the investigation of different BN^•+^–(H_2_O)_*n*_ clusters. Specifically, Chatterjee and Dopfer first measured the high-resolution IR spectrum of BN^•+^–H_2_O via mass-selected IRPD spectroscopy (Figure 1a).^9^ By performing dispersion-corrected hybrid density functional theory (DFT) calculations, the observed IRPD spectrum was attributed to two isomers with the bifurcated C–H···O hydrogen bonds between water and either ortho/meta (o/m) or meta/para (m/p) adjacent C–H groups (Figure 1a), although the broadband feature of the C-band around 3080 cm^–1^ was not reproduced.^9^ Recently, Mason et al. employed a mass-selected ion mobility (MSIM) system to examine the equilibrium abundances of BN^•+^–(H_2_O)_*n*_ (*n* = 1–7) at various temperatures and water vapor pressures (Figure 1b).^7^ They found that BN^•+^–(H_2_O)_*n*_ (*n* = 1–3) species have only trace amounts. Based on the calculations at a hybrid DFT level without dispersion correction, the absence of BN^•+^–(H_2_O)_*n*=1–3_ was attributed to a water-assisted proton transfer process. Specifically, when *n* ≥ 4, a significant stable “distonic” cation that consists of a deprotonated radical ^•^C_6_H_4_CN and a protonated water cluster H­(H_2_O)_*n*_^+^ forms, which rapidly depletes the BN^•+^–(H_2_O)_*n*=1–3_ clusters (Figure 1b).^7^

**Figure 1:**
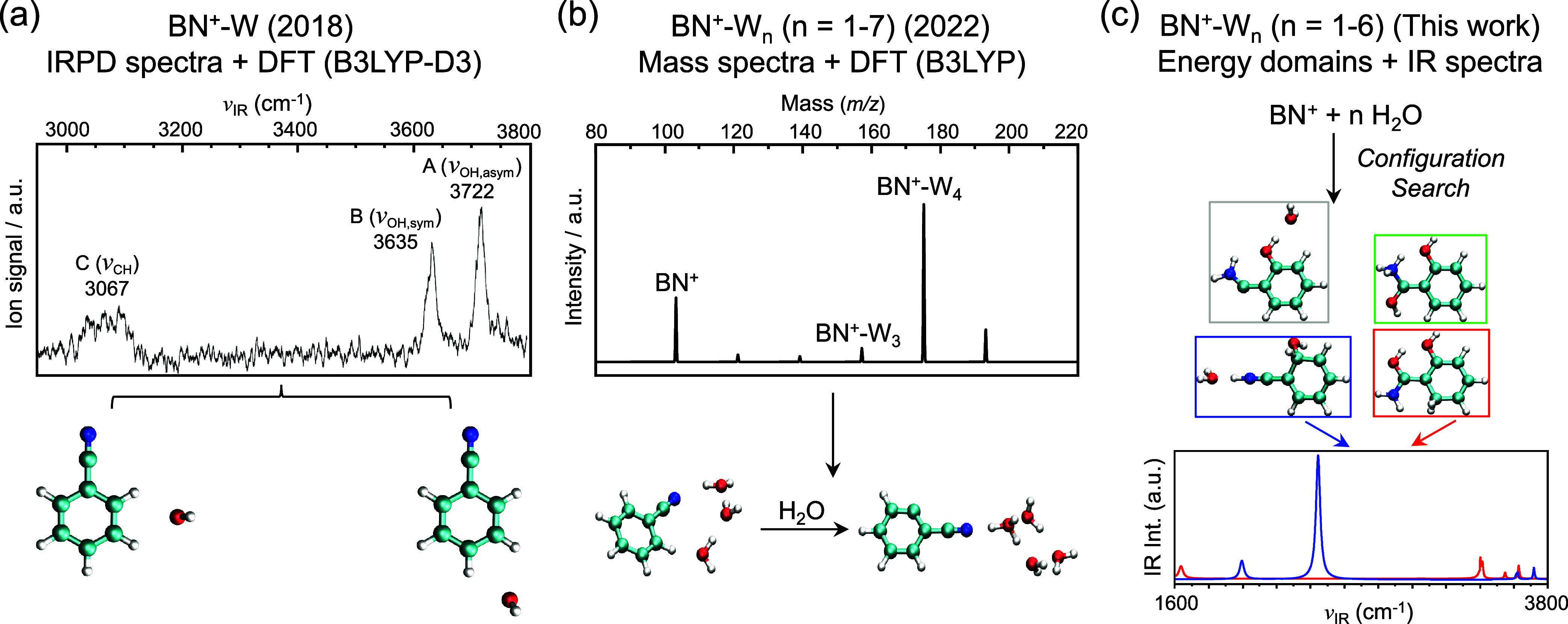
Methods of investigating the hydration of BN^•+^. (a) IRPD spectrum of BN^•+^–H_2_O. Frequency and assignment of each band are marked. The two determined isomers (m/p and o/m) contribute to all three observed bands. Reproduced with permission from ref 9. Copyright 2018 AIP Publishing. (b) Mass spectrum of BN^•+^–(H_2_O)_*n*_ (*n* = 1–7) at equilibrium (300 K, 0.25 Torr water vapor pressures). A water-assisted hydrogen abstraction process was suggested. Reproduced with permission from ref 7. Copyright 2022 American Chemical Society. (c) Exhaustive configuration search for BN^•+^–(H_2_O)_*n*_ (*n* = 1–6) and simulated IR spectra in the present work. Four kinds of low-lying species are identified. Publication years are indicated in parentheses.

It should be noted that due to the high flexibility of hydrogen-bond networks,^12^ a specific configuration can hardly represent the overall properties of hydrated clusters.^13,14^ To provide decisive insights into the structural evolution of BN^•+^–(H_2_O)_*n*_, detailed investigations of the potential energy surface (PES) and “finger-print” vibrational features based on high-accuracy theoretical methods are urgently needed. In this work, we applied a stepwise configuration search protocol to exhaustively investigate the potential products and corresponding reaction pathways in the sequential hydration of BN^•+^ (Figure 1c). With two newly identified low-lying isomers at the high-accuracy theoretical level, the population-averaged IR spectrum of BN^•+^–H_2_O became quantitatively in agreement with the experimental observation. For clusters with *n* ≥ 2, numerous isomers were predicted, which can be categorized into several separate energy domains. The calculated energy barriers between different categories revealed a kinetically stable species, which is responsible for the trace abundance of BN^•+^–(H_2_O)_*n*_ (*n* = 1–3) recorded in the mass spectra. Furthermore, the theoretical IR spectra of all isomers were simulated, providing decisive vibrational signatures for the kinetically stable and most stable species.

## 2 Computational Details

The configuration search in this work was performed by the algorithm of partitioning the whole system into monomers and generating initial structures via randomly emitting specified numbers of monomers, as implemented in Molclus.^15^ The initial candidates were prescreened with the semiempirical method PM6-D3H4 ^16,17^ as implemented in MOPAC.^18^ Geometries within an energy window of 15 kcal/mol were retained and further optimized at the B3LYP-D3­(BJ)/6-31G­(d,p) level^19–21^ using the Gaussian 16 suite of programs.^22^ Upon the optimized geometries, vibrational analyses were performed to obtain thermodynamic corrections. Configurations with their relative energies below 3.6 kcal/mol from the global minimum were retained. In cases where new bonding patterns emerged during the search, these patterns were extracted as new monomers for another round of search, while constraints were applied to the original monomers to keep their connectivity. Further optimization and vibrational analysis were conducted for the structures that remained after the first two steps of screening at the ωB97X-D4/def2-TZVPPD level^23–27^ with ORCA 5,^28^ where the resolution-of-the-identity (RI) approximation^29,30^ was applied for Coulomb integrals with the chain-of-spheres exchange (COSX) approximation^31,32^ for exchange integrals. Finally, to obtain highly accurate energetics and IR spectra, all isomers with Boltzmann weights greater than 3% at room temperature were optimized at the XYGJ-OS/may-cc-pVTZ level.^33–40^ Then, the IR spectra were calculated analytically for the configurations at the same level using a locally modified version of Gaussian 09.^41^ In the meantime, electronic energies were further refined at the DLPNO-CCSD­(T)/aug-cc-pVTZ level^37–39,42–44^ with the “tightPNO” setting and the RI approximation for four electron integrals^45,46^ and correlation energies^47,48^ to calibrate the DFT energies.

For transition states that connect identified isomers, model systems were first optimized and validated by intrinsic reaction coordinate (IRC) calculations at the B3LYP-D3­(BJ)/6-311G­(d,p) level^19–21,49,50^ using Gaussian 16 ^22^ to help identify the reaction region. During the configuration search, atoms in the reaction region were not allowed to move until the final screening step,^51^ i.e., the ωB97X-D4/def2-TZVPPD step, where all constraints were released. The nature of the transition states was then verified by the imaginary vibrational mode of the configurations that were found.

The Gibbs free energies of all identified stationary configurations were calculated based on corresponding frequency analyses, where the thermodynamic corrections were evaluated using Grimme’s quasi-RRHO model^52^ as implemented in Shermo^53^ at 298 K and 1 atm unless otherwise stated. A uniform scaling factor of 0.9487 determined by fitting the most intense experimental IR band of BN^•+^–H_2_O was applied to the calculated fundamental frequencies. Reaction rate constants were computed with the help of KiSThelP.^54^ All wave function analyses were carried out using Multiwfn.^55,56^

## 3 Results and Discussion

### 3.1 BN^•+^···H_2_O Isomers and IR Spectra

The measured high-resolution IR spectrum^9^ provides an excellent benchmark for current investigations. Through an exhaustive configuration search at the theoretical level as high as the XYGJ-OS/may-cc-pVTZ level, the free energy differences of all five identified isomers are in the region of 1.5 kJ/mol (Figure 2a). It should be emphasized that these five isomers are all the local minima obtained at the XYGJ-OS/may-cc-pVTZ level and other isomers were excluded by frequency analysis. Among the obtained five structures, three previously reported isomers, i.e., the aforementioned m/p and o/m configurations as well as the so-called π configurations in which H_2_O is located above the aromatic plane,^9^ have been determined. It should be noted that, in the latter isomer, the H_2_O molecule plane almost aligns with the *C*_2_-axis of the BN^•+^ moiety (with a dihedral angle of around 8°, Figure 2a), which is in contrast to the nearly perpendicular angle between them in the previously reported configuration.^9^ In fact, the perpendicular configuration has one imaginary frequency at the high-level vibrational analysis (48.7 cm^–1^), and is thus assigned as a transition state. In the aligned configuration, the shortest O···C bond length between H_2_O and BN^•+^ is 2.80 Å, which is 0.04 Å longer than that in the perpendicular configuration. Further independent gradient model based on Hirshfeld partition (IGMH) analysis^57^ indicates that the above H_2_O has a strong nonbond interaction with the 1-position carbon atom (C_1_) rather than the whole π electrons (Figures 2a and S1). Thus, we renamed the aligned configuration as the C_1_-isomer hereafter.

**Figure 2:**
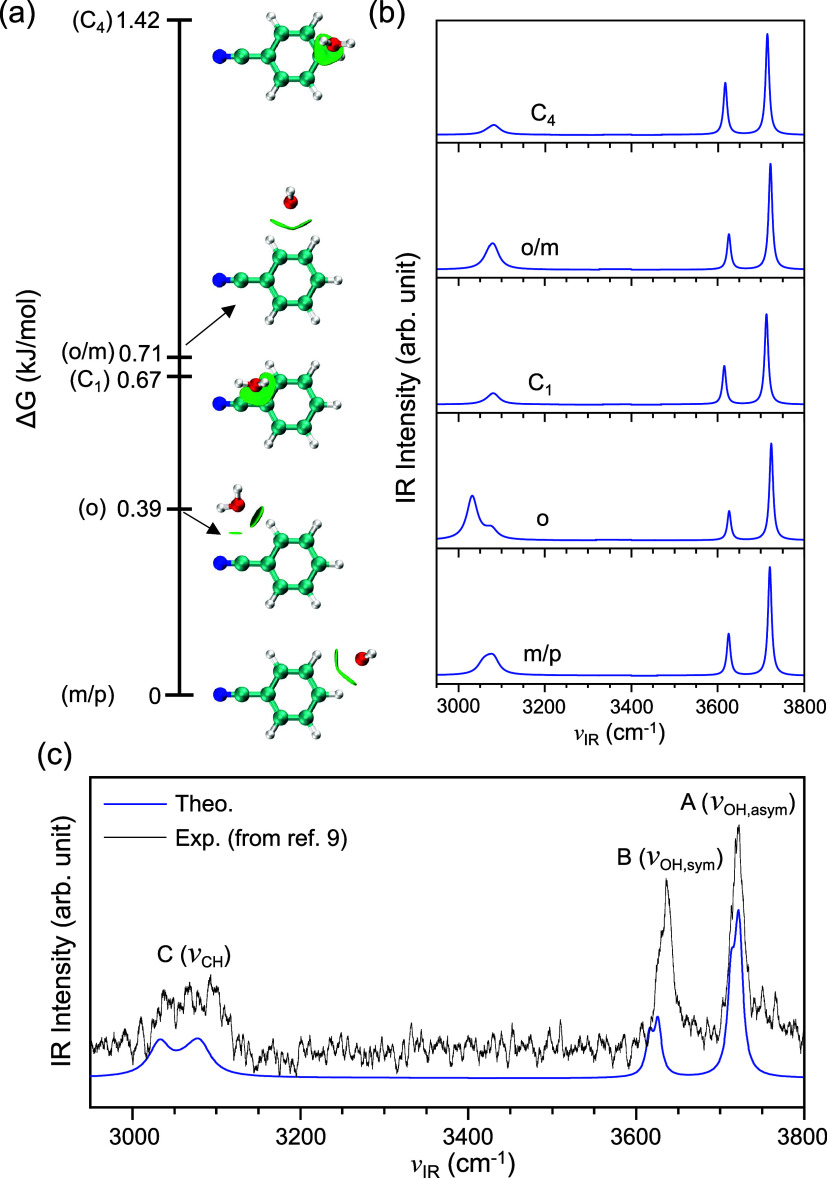
Low-lying configurations and IR spectra of BN^•+^–H_2_O. (a) Identified stationary structures with graphical representations of the weak interactions^57^ between BN^•+^ and H_2_O moieties (green isosurfaces). Labels in parentheses indicate the binding site. (b) Simulated IR spectra of identified configurations. (c) Calculated thermal-averaged IR spectrum (blue line) compared with the experimental IRPD spectrum^9^ (black line). The experimental spectrum is reproduced with permission from ref 9. Copyright 2018 AIP Publishing.

Besides these isomers, two new structures at the stationary point were further identified in the present work (Figure 2a). One is the o-isomer, where the H_2_O molecule interacts with the hydrogen atom at the ortho position. In previous theoretical simulations, the o-isomer was predicted as a transition state, where H_2_O is in the plane of the BN^•+^ moiety. However, in the new o-isomer, the dihedral angle between the H_2_O molecule plane and the BN^•+^ plane is around 30°. At the high theoretical level of XYGJ-OS/may-cc-pVTZ, all calculated frequencies of the new o-isomer are positive, confirming that this structure is indeed a stationary configuration. Another new structure is the C_4_-isomer, where H_2_O above the aromatic plane interacts with the 4-position carbon atom in the BN^•+^ fragment (Figure 2a). In addition, the H_2_O plane exactly aligns with the *C*_2_-axis of the BN^•+^ moiety, which suggests a *C_S_* point group, differing from that of the C_1_-isomer (Figure 2a). Further IGMH analysis^57^ reveals that the m/p-, o-, and o/m-isomers include unconventional carbon-based ionic hydrogen bond(s), while the C_1_- and C_4_-isomers contain carbon–oxygen nonbonding interactions (Figure 2a).

At the high theoretical level, the predicted energy order and differences between the isomers are different from those at the conventional hybrid functional level, although the most stable configuration is the m/p isomer at all levels. Specifically, the second most stable configuration at the XYGJ-OS/may-cc-pVTZ level is the newly identified o-isomer, rather than the C_1_-isomer predicted at the B3LYP-D3/aug-cc-pVTZ level.^9^ In addition to the change in energy order, the energy differences between the isomers at a higher theoretical level are much smaller than those obtained at the conventional level. For instance, at the DLPNO-CCSD­(T)/aug-cc-pVTZ//XYGJ-OS/may-cc-pVTZ level, the relative energy of the o/m-isomer with respect to the stalest m/p-isomer is only 0.71 kJ/mol, which is 0.80 kJ/mol (53%) lower than the value at the B3LYP-D3/aug-cc-pVTZ level.^9^ This small energy difference was confirmed by calculations at the canonical CCSD­(T)/may-cc-pVTZ//XYGJ-OS/may-cc-pVTZ level, which gives a very close relative energy of 0.50 kJ/mol (Table S1).

The accurate energy calculations and the newly identified isomers are crucial for the quantitative reproduction of the observed high-resolution IR spectrum of BN^•+^–H_2_O. In previous theoretical simulations, the low-frequency side of the C-band, i.e., the C–H stretching modes, was absent in the calculated spectra of the m/p- and o/m-isomers. This result should be attributed to the distributed interaction across the bifurcated C–H···O hydrogen bonds in these two isomers. In the newly identified o-isomer, the water solely interacts with the ortho-position C–H bond, resulting in a significant red shift and enhanced IR intensity of the C–H stretching (Figure 2b). It should be noted that the chiral nature of the o-isomer doubles its population, leading to its contribution (27.5%) being even larger than that of the most stable m/p-isomer (18.0%) under ambient temperature. Consequently, the full width at the half-maximum (fwhm) of the C-band in the theoretically predicted population-averaged IR spectrum is around 50 cm^–1^, which closely matches the experimental counterpart of 80 cm^–1^ (Figure 2c). The newly identified isomers and accurate free energies are also important for the O–H stretching modes, i.e., the A-band and B-band. Specifically, the enhanced intensity and red shift of the symmetric (B-band) and antisymmetric (A-band) O–H stretching in the C_4_-isomer are important for the shoulder peaks in the low-frequency side of these bands. Meanwhile, the o-isomer is responsible for the main peaks in the high-frequency side, particularly for the A-band. Thus, we can conclude that a comprehensive configuration search as well as accurate free energy and spectral calculations in the present work are essential for quantitatively interpreting the experimental measurements of the BN^•+^–H_2_O cluster. It should be noted that although many high-level methods can describe the thermodynamics of hydration accurately (Table S2), the current level is one of the few levels that can quantitatively reproduce the experimental IR spectrum (Figures S2 and S3).

### 3.2 Energy Domains of BN^•+^–(H_2_O)_*n*≥2_

For clusters containing more than one water molecule, a comprehensive structure search becomes more vital. For instance, except the previously suggested distonic ^•^C_6_H_4_CN···H^+^(H_2_O)_*n*_ species,^7^ four additional species with lower energies have been identified in the present work (Figure 3). The newly discovered species can be classified into two categories. In one category, i.e., Species 1, a direct ortho-hydroxylation occurs at the BN ring with a proton transfer. As a result, the *ortho*-carbon changes its hybridization from sp^2^ to sp^3^. By definition, Species 1 also belongs to the distonic group because the radical locates at the BN moiety, while the positive charge is distributed in the protonated water clusters. In Species 1, the hydroxyl group and the electron-rich nitrogen atom can both act as hydrogen-bond acceptors, providing anchoring sites for unique ring-like hydrogen-bonded connections with protonated water clusters. On the other hand, all other species are not distonic and should be assigned to the other category, in which the radical and positive charge are all located at the BN moiety. Specifically, Species 2 arises from a water-assisted C–H migration^58,59^ of Species 1. As a result, two hydrogen atoms are added to the nitrogen atom, forming an NH_2_ group. In the meantime, the hybridization of the carbon in CN changes from sp to sp^2^, while the hybridization of the *ortho*-carbon reverts to sp^2^. Species 3 and 4 are produced via a nucleophilic addition of water to the α-carbon of the amino group of Species 2, followed by a further water-assisted proton transfer. Depending on which site receives the proton, an −NH_3_^+^ group is generated (Species 3) or the other *ortho*-carbon undergoes a sp^2^ to sp^3^ transformation (Species 4).

**Figure 3:**
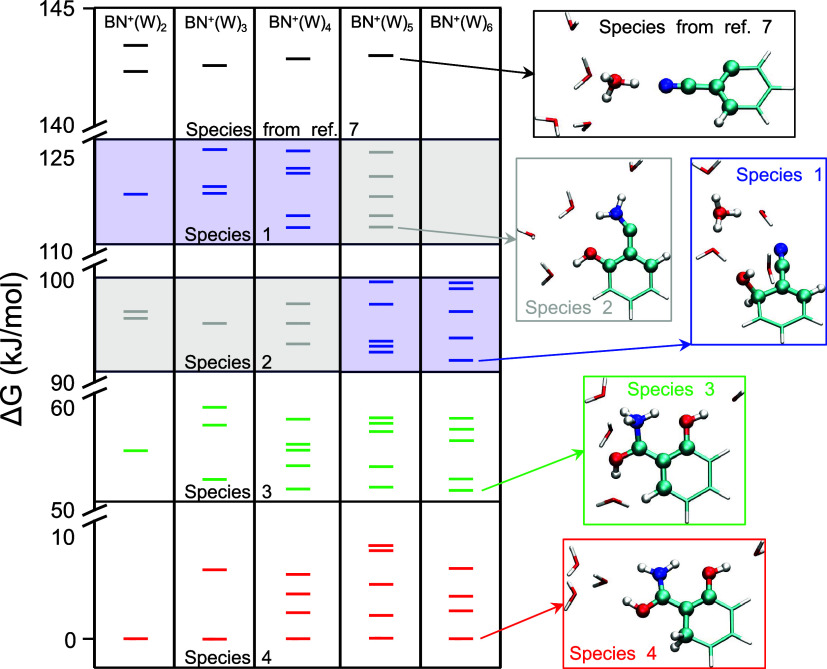
Calculated energy domains of BN^•+^–(H_2_O)_*n*_ (*n* = 2–6) and representative structures in each domain. The isomers belonging to the distonic species determined in ref 7 are included for comparison.

Due to either the high flexibility of hydrogen-bond networks (Species 1) or the presence of multiple hydrogen-bond donors (Species 2–4), each species contains several nearly isoenergetic configurations, as shown in Figure 3. Interestingly, intramolecular hydrogen bonding does not significantly alter the affinity for hydrogen-bond acceptors of the hydroxyl and amino groups, leading to degenerate energies for the two isomers of Species 2 at *n* = 2. For *n* ≥ 4, the number of nearly isoenergetic configurations significantly increased. Specifically, the number of isomers within the relative energy window of 10 kJ/mol is greater than or equal to 4, except for Species 2 of BN^•+^–(H_2_O)_4_.

To further reveal the underlying mechanism of the newly determined low-lying isomers, electronic structure analysis was performed for BN^•+^ and BN^•+^–(H_2_O)_3_ as a representative example. Mayer bond order^60^ analysis of BN^•+^ shows that the bond orders of C_1_–C_2_ (C_6_–C_1_), C_2_–C_3_ (C_5_–C_6_), and C_3_–C_4_ (C_4_–C_5_) are 1.09, 1.43, and 1.16, respectively, confirming the localized C=C bond and thus weak conjugated hexadiene-like structure of BN^•+^. Hydroxyl addition to C_2_ in Species 1 withdraws π electrons from C_2_, giving bond orders of C_1_–C_2_ and C_2_–C_3_ of 0.90 and 1.00, respectively. The remaining bond orders become 1.50, 1.14, 1.22, and 1.39 for C_3_–C_4_, C_4_–C_5_, C_5_–C_6_, and C_6_–C_1_, respectively. This result indicates the delocalization of π electrons among C_3_, C_4_, C_5_, C_6_, and C_1_, bringing extra stabilization energy to Species 1. Species 2 and Species 3 restore a formal aromatic ring structure. On the other hand, Species 4 possesses similar characteristics to Species 1, i.e., experiencing a disruption of conjugation at the ortho position. However, in the former, the OH group participates in the π-electron conjugation, which helps it become the most stable species. Therefore, each species is located in a well-separated energy domain, as shown in Figure 3. Furthermore, the calculated multicenter bond index (MCI)^61^ of BN is around 0.004, 0.028, 0.027, and 0.002 for Species 1 to 4, respectively. In contrast, the MCI values of C_6_H_5_CN and C_6_H_5_CN^•+^ are 0.042 and 0.021, respectively, suggesting that aromaticity has little influence in evaluating the stability of the newly identified species.

### 3.3 Isomerization Pathways of BN^•+^–(H_2_O)_*n*≥2_

Because several low-energy species have been identified, it is necessary to investigate the kinetic behaviors between them to gain insight into the dominant species of BN^•+^–(H_2_O)_*n*_ under different conditions. To this end, we trace the isomerization pathways from conventional radical cation–water complexes to newly identified species (Figure 4). Figure 4a shows the calculated free energy profile for the isomerization of BN^•+^–(H_2_O)_2_. The initial state is the isomer where two water molecules are located at the ortho and m/p-position, respectively. Through a water-assisted nucleophilic addition with a negligible barrier of 0.3 kcal/mol, an OH group is added to the *ortho*-carbon. Meanwhile, proton transfer subsequently occurs, yielding P_2_^1′^ (hereafter “P” represents product, subscript indicates the number of water molecules, and superscript indicates the Species index). Nevertheless, the ring consisting of *ortho*-OH, H_3_O^+^, and cyano groups exhibits significant strain. Therefore, the strong electrostatic attraction of the cyano nitrogen drives a further proton transfer, leading to a ring-opening process with an energy barrier of 3.5 kcal/mol. Although the ring is absent, we still classify the product as P_2_^1^ due to its characteristic *ortho*-carbon and stability.

**Figure 4:**
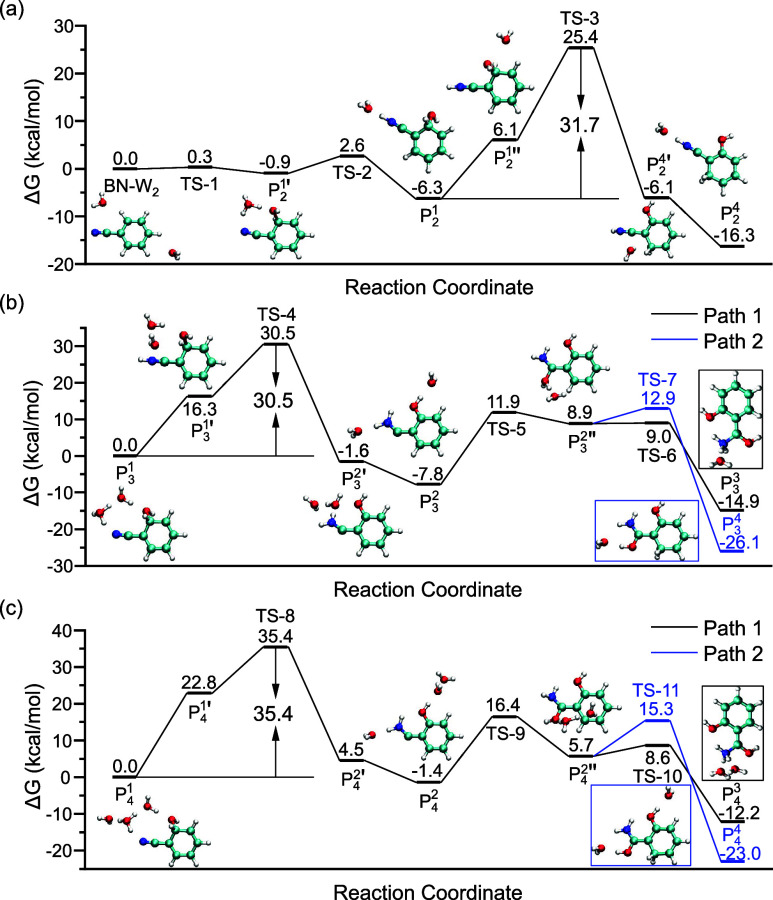
Calculated reaction free energy profiles connecting different species for BN^•+^–(H_2_O)_*n*=2–4_. (a) Reaction pathway from the conventional BN^•+^–(H_2_O)_2_ complex to Species 4 via the distonic Species 1. (b) Reaction pathways from Species 1 to Species 3 and 4 for BN^•+^–(H_2_O)_3_ via Species 2. (c) Reaction pathways from Species 1 to Species 3 and Species 4 for BN^•+^–(H_2_O)_4_. Optimized structures of representative local minima are depicted. The free energy barriers of the rate-determining steps are labeled. “TS” represents the transition state.

P_2_^1^ can be further converted to other species. Specifically, with a change in the position of H_2_O in P_2_^1^, P_2_^1″^, i.e., another isomer in Species 1 with a relatively high free energy of 6.1 kcal/mol, is generated. IRC calculations show that through TS-3, P_2_^1″^ can be covered to P_2_^4′^ (a high-energy isomer of Species 4), in which the hydrogen on the sp^3^ *ortho*-carbon migrates to the other sp^2^ *ortho*-carbon, exchanging the hybridization modes of the two sites. It should be noted that the overall free energy barrier from P_2_^1^ to P_2_^4′^ is as high as 31.7 kcal/mol, which is inaccessible under conventional reaction conditions. After facile movement of the bound water molecule, P_2_^4′^ can easily reach its most stable structure, i.e., P_2_^4^ (Figure 4a). Once P_2_^4^ is generated, the most stable P_3_^4^ isomer would be readily produced in the presence of free water molecules (Figure S4).

Because an extra water molecule is added, in BN^•+^–(H_2_O)_3_, the strain of the ring consisting of the *ortho*-OH, H­(H_2_O)_2_^+^, and cyano groups is significantly decreased. Thus, the ring structure is retained in the most stable configuration in Species 1, i.e., P_3_^1^ (Figure 4b). In a manner similar to that of BN^•+^–(H_2_O)_2_, P_3_^1^ would convert to P_3_^2^ via TS-4 with an overall free barrier of 30.5 kcal/mol, where P_3_^1′^ and P_3_^2′^ are intermediates between them (Figure 4b). Detailed analysis of the calculated IRC trajectories reveals that, in the reaction pathway, two water molecules play complementary roles. Specifically, one water abstracts C–H and another delivers the proton transferred through the hydrogen-bond network to nitrogen, which highlights the regulatory role of hydrogen-bond bridging in proton transfer reactions. Isomerization of P_3_^2^ includes another nucleophilic addition of water to the α-carbon of the amino group, producing an intermediate of P_3_^2″^. The reaction barrier is 19.7 kcal/mol. P_3_^2″^ would undergo a pathway divergence depending on the direction of proton transfer in TS-6 and TS-7, generating the kinetic product of P_3_^3^ and the thermodynamic product of P_3_^4^, respectively. The overall reaction barrier from P_3_^2^ to P_3_^3^ is the same as that for P_3_^2″^. On the other hand, the overall barrier from P_3_^2^ to P_3_^4^ is 20.7 kcal/mol (Figure 4b).

For BN^•+^–(H_2_O)_*n*_ with *n* ≥ 4, the extra water molecules could surround the kernel of the protonated water cluster via hydrogen bonding. Thus, the energy profiles of their isomerization are quite similar to those of BN^•+^–(H_2_O)_3_. For instance, the energy barrier from P_4_^1^ to P_4_^2^ is 35.4 kcal/mol (Figure 4c), of which the increase should be attributed to the extra-strong hydrogen bond between the newly added H_2_O and the H_3_O^+^ moiety. On the other hand, the overall free barriers of P_4_^2^ to kinetic product P_4_^3^ and thermodynamic product P_4_^4^ are both 17.8 kcal/mol.

We can observe that in all cases, the energy barrier from Species 2 to Species 3 or Species 4 is around 20 kcal/mol, which is significantly lower than that from Species 1 to Species 2 (over 30 kcal/mol). As a result, high-energy Species 2 (with respect to Species 3 and 4) exhibits intermediate character on the potential energy surfaces and thus becomes less important. Test calculations show that the barrier from the high-energy distonic isomers identified in ref 7 to Species 1 is less than 24 kcal/mol, suggesting that the former could easily convert to Species 1 at ambient temperatures. According to the reaction rate constant calculations^62^ incorporating tunneling coefficients^63^ (Tables S3 and S4), the lifetime of Species 1 would exceed 10^8^ s (around 30,000 h) at such a low temperature. When temperatures are high enough to overcome the energy barrier between Species 1 and 2, all subsequent isomerizations can readily proceed, leading to the final product as the most stable Species 4. In other words, there is a separate energy domain between Species 1 and other species. As a result, the dominant isomers of BN^•+^–(H_2_O)_*n*_ under the low (<400 K) and high (>700 K) temperatures are Species 1 and Species 4, respectively.

### 3.4 Equilibrium Population of BN^•+^–(H_2_O)_*n*≤6_ in Mass Spectrometry

To validate the proposed dominant isomers of BN^•+^–(H_2_O)_*n*_, the experimentally measured mass spectra at ambient temperatures and different water vapor pressures^7^ were quantitatively reproduced by calculating the thermodynamic equilibrium populations under the same conditions (Figure 5). By adopting newly identified distonic Species 1 for all BN^•+^–(H_2_O)_*n*≤6_ isomers, the experimentally observed abundance gap between *n* = 3 and 4 is reproduced under different conditions. In this context, when the temperatures are higher than 270 K, calculated free energies suggest that the BN^•+^–(H_2_O)_4_ isomers are dominant, while BN^•+^–(H_2_O)_5_ is the most intensive one at the lower temperatures of 268 and 254 K. The missing BN^•+^–(H_2_O)_*n*≤3_ clusters can be attributed to the strong hydrogen binding of the fourth water molecule to the H_3_O^+^ moiety, which dramatically stabilizes the clusters with *n* ≥ 4. Specifically, the calculated enthalpy from *n* = 3 to 4 is −15.9 kcal/mol, which is characteristic of an exceptionally strong charged hydrogen bond.^64^ Instead, for *n* = 4–5 and 5–6, the computed enthalpies were −12.0 and −10.0 kcal/mol, respectively, agreeing well with the experimental measurements of −11.3 ± 1.0 and −11.0 ± 1.0 kcal/mol.^7^

**Figure 5:**
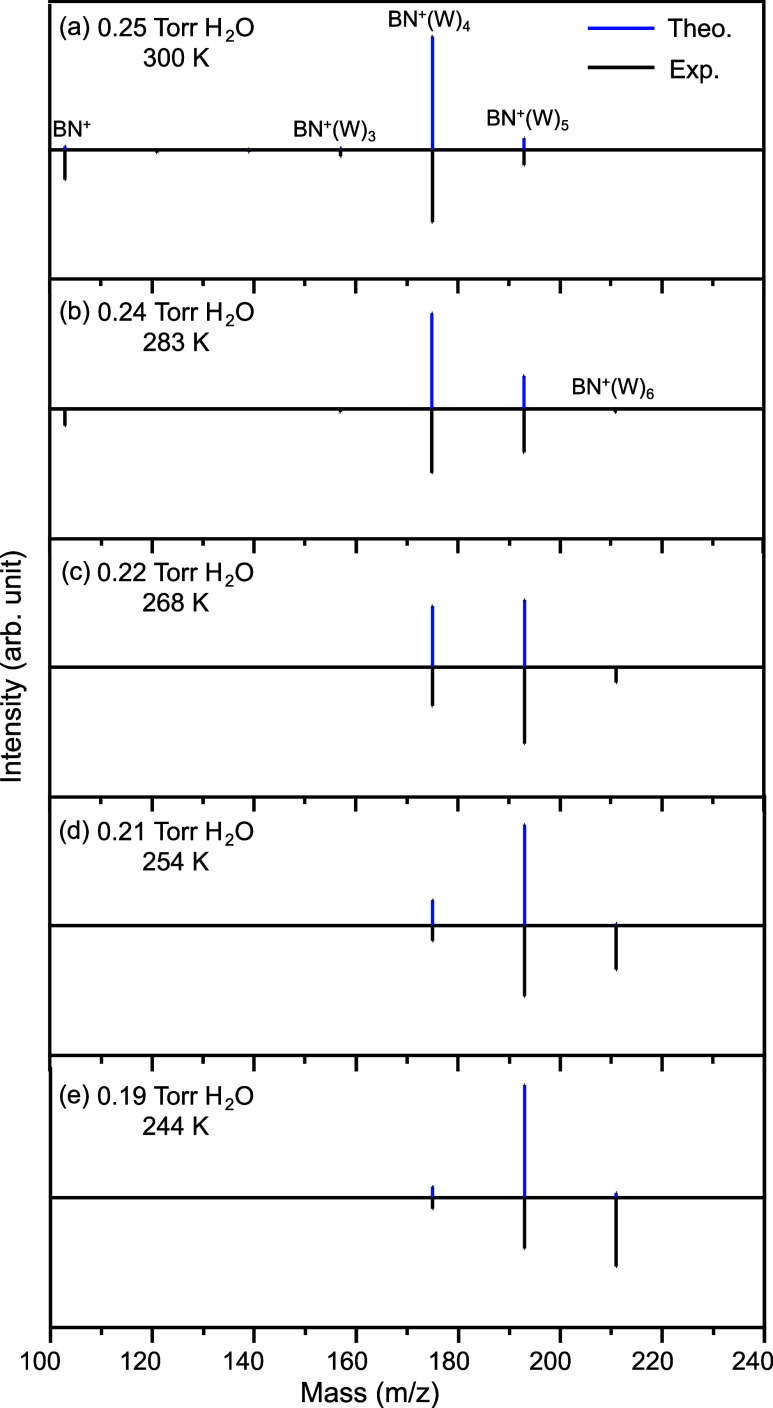
Calculated relative abundances of BN^•+^–(H_2_O)_*n*_ (*n* ≤ 6) at equilibrium of Species 1 (blue bars) under various conditions of (a) 0.25 Torr H_2_O, 300 K; (b) 0.24 Torr H_2_O, 283 K; (c) 0.22 Torr H_2_O, 268 K; (d) 0.21 Torr H_2_O, 254 K; and (e) 0.19 Torr H_2_O, 244 K. The experimentally measured MSIM mass spectra (black bars) are depicted for comparison, which is reproduced from ref 7. Copyright 2022 American Chemical Society.

When the temperature is further reduced to 244 K, the experimentally recorded mass spectrum showed that the most abundant cluster is BN^•+^–(H_2_O)_6_. However, the theoretical calculations underestimate the population of BN^•+^–(H_2_O)_6_ as shown in Figure 5. This result should be attributed to the accuracy of the theoretical method itself as well as the possible incompleteness of the configuration search for large clusters. To examine the effect, we find that in most cases, an adjustment of calculated free energies within the chemical accuracy (1 kcal/mol) could exactly reproduce the measured relative abundances under most conditions (Figure S5). In contrast, our test calculations show that, if the distonic species identified in ref 7 were adopted, no hydrated cluster could be observed under the experimental conditions because these species do not have adequately low free energies (Figure S6). Moreover, when the temperature is raised such that Species 4 becomes the dominant cluster, the observed abundance gap in the experiment will disappear. In these scenarios, the most abundant product will be BN^•+^–(H_2_O)_3_ instead (Figure S6). All of these results confirm that the newly identified distonic Species 1 is indeed the dominant species observed under the experimental conditions for BN^•+^–(H_2_O)_*n*≥2_. It should be stressed again that no new structural motifs were found during the configurational search with *n* > 4, which is consistent with the experimentally observed trends of the relative abundance of the hydrated clusters with *n* > 3. In other words, it is expected that the species of *n* = 7 would be the same as those for smaller values of *n*. Moreover, we can conclude that Species 1 remains dominant at *n* = 7 in the experimental mass spectra because this species becomes more and more kinetically stable with the increase of the value of *n*, as shown in Figure 4.

### 3.5 Observable IR Spectra of BN^•+^–(H_2_O)_*n*≥2_

As in the case of BN^•+^–H_2_O, measurable IR spectra that have vibrational fingerprints can provide informative structural details, thereby conclusively confirming the dominant isomers for *n* ≥ 2 under different conditions. To this end, we calculate the thermal-averaged IR spectra of BN^•+^–(H_2_O)_*n*_ (*n* = 2–6) at a high theoretical level of XYGJ-OS/may-cc-pVTZ (Figure 6). As can be seen, kinetically stable distonic Species 1 has a characteristic band that appears around 2400 cm^–1^. This band originates from the unique proton shuttling between H_3_O^+^ and the water molecule that forms a hydrogen bond with it (or between water and the protonated cyano group for *n* = 2), coupled with symmetric stretching of the water molecule(s). The only exception is BN^•+^–(H_2_O)_3_, in which proton shuttling strongly couples with the scissoring of the two water molecules. As a result, the frequency of this band significantly red-shifts to around 1900 cm^–1^. Owing to the characteristic ring structure as well as the distonic feature in Species 1, various bands appear in the region between 1700 and 2700 cm^–1^ (Figure 6).

**Figure 6:**
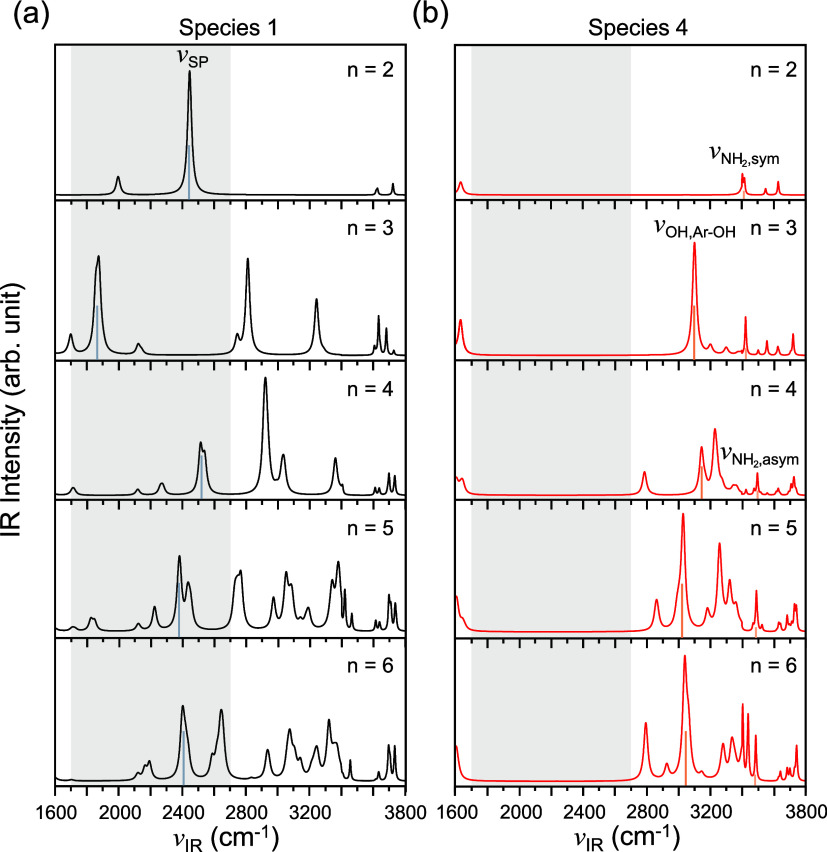
Simulated thermal-averaged IR spectra of kinetically stable Species 1 (a) and thermodynamically stable Species 4 (b) of BN^•+^–(H_2_O)_*n*_ (*n* = 2–6) (from top to bottom) at room temperature. Characteristic absorption bands are marked by vertical lines, and “SP” denotes shared proton.

At high temperatures, Species 4 becomes dominant owing to its thermodynamic stability, which gives distinguishable IR spectra to Species 1 (Figure 6). For BN^•+^–(H_2_O)_2_, the most intense band occurs around 3400 cm^–1^, which originates from the coupling between the stretching of the O–H bond in the hydroxyl group bonded to the original cyano carbon and the N–H bonds in the amino group. Since the hydrogen atoms involved are less charged than the proton in Species 1, the two modes do not change the dipole moment as much, thus exhibiting much weaker absorption. For clusters with *n* ≥ 3, owing to the stretching of the O–H bond in the phenol group and the N–H bonds in the amino group, two characteristic bands are exhibited around 3100 and 3500 cm^–1^, respectively. Since the ring structure associated with the protonated water cluster is absent in Species 4, no IR bands appear in the region between 1700 and 2700 cm^–1^ across all cluster sizes studied, providing a clear diagnostic feature for distinguishing the two dominant species.

### 3.6 Summary of the Structural Evolution of BN^•+^–(H_2_O)_*n*_

Finally, the structural evolution of BN^•+^–(H_2_O)_*n*_ with different values of *n* during sequential hydration is summarized in Figure 7. When *n* = 1, BN^•+^ binds a single H_2_O via weak noncovalent interactions, forming a conventional radical cation–water complex. At ambient temperatures, upon collision with a second water molecule, water-assisted nucleophilic addition of H_2_O to the *ortho*-carbon occurs with subsequent proton transfer via the other H_2_O to the nitrogen of the cyano group, producing a sp^3^ hybridized *ortho*-carbon. Upon further hydration (*n* ≥ 3), the proton is recaptured by the surrounding water molecules, yielding a kinetically stable distonic isomer featuring a hydrogen-bonded ring comprising the radical and a protonated water cluster. At elevated temperatures, the kinetically stable products undergo water-assisted hydrogen migrations. For *n* = 2, the hydrogen on the *ortho*-carbon with an OH group migrates to the other *ortho*-carbon at temperatures higher than 700 K. For *n* ≥ 3, the required temperatures could be as low as 600 K. At such temperatures, the nitrogen atom in the cyano group is first hydrogenated, forming an ammonia intermediate. Then, the intermediate can readily overcome the subsequent barriers for water-assisted nucleophilic addition, achieving the theoretically most stable species.

**Figure 7:**
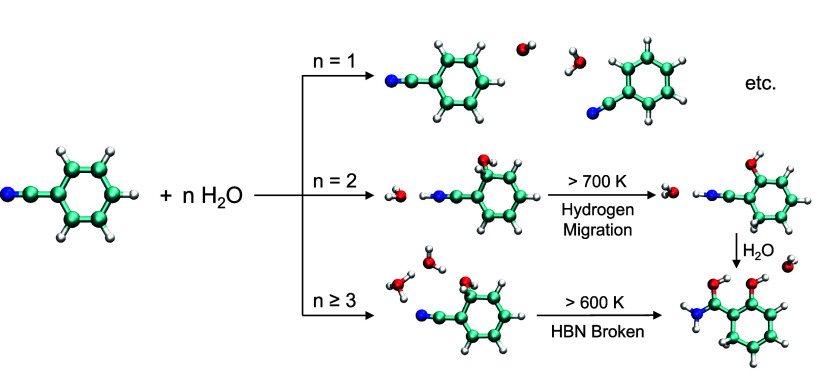
Summary of dominant isomers in the sequential hydration of BN^•+^ under different conditions. “HBN” denotes a hydrogen-bond network.

## 4 Conclusions

In summary, we comprehensively explore the structural evolution for BN^•+^–(H_2_O)_*n*=1–6_ clusters and the corresponding IR spectra at the high-accuracy theoretical level. For BN^•+^–(H_2_O), new isomers have been identified by an exhaustive configuration search. Among them, the chiral o-isomer becomes the most abundant population under ambient conditions, which plays a key role in the quantitative reproduction of the experimentally measured IRPD spectrum of BN^•+^–H_2_O. For *n* ≥ 2, four kinds of structures have been identified, which can be classified into two categories, i.e., distonic and convectional radical cation-water complexes, separated by an energy barrier as high as around 30 kcal/mol. The former isomers are kinetically stable at ambient temperatures and have much lower energies than the previously identified distonic structures. On the other hand, the latter isomers are thermodynamically stable, which can exist under high temperatures. The newly determined distonic complexes contain a characteristic ring that consists of a protonated water cluster, cyano group, and hydroxyl substituent for *n* ≥ 3. The protonated water cluster provides a strong hydrogen bonding site, which significantly stabilizes the distonic complexes with *n* ≥ 4 and thus is responsible for the experimentally observed abundance gap of mass spectra at ambient temperatures. The unique ring structure corresponds to characteristic O–H stretching modes located in the low-frequency region, providing a diagnostic IR signature for distinguishing kinetically and thermodynamically stable isomers of BN^•+^–(H_2_O)_*n*≥2_. Overall, the findings in the present work not only advance our understanding of the microscopic structures of hydrated polar group-substituted aromatic complexes but also shed light on the interactions between other organic radicals and solvents.

## Supplementary Material



## Notes

Details on the accuracy benchmark of the used theoretical levels, together with the tables summarizing the reaction kinetics data of the rate-determining steps and figures depicting the aromaticity of BN and BN^•+^, the isomerization pathway for P_2_^4^ in the presence of free water and the relative abundance of other species at ambient temperature (PDF)
