# Supplementary material for: Separated Energy Domains in the Sequential Hydration of a Benzonitrile Radical Cation
Source: Precis Chem. 2025 Dec 31;4(4):388–97. doi: 10.1021/prechem.5c00294 (PMC13126368; doi:10.1021/prechem.5c00294)
Supplement: Supplementary file 1 [file pc5c00294_si_001.pdf]

Supporting Information for Separated Energy Domains in the Sequential Hydration of a Benzonitrile Radical Cation

Bingzheng Wu,† Shirong Wang,† Xin Xu,*,†,‡ and Sai Duan*,†,‡

†*State Key Laboratory of Porous Materials for Separation and Conversion, Shanghai Key Laboratory of Molecular Catalysis and Innovative Materials, MOE Key Laboratory of Computational Physical Sciences, Research Center for Chemical Theory, Department of Chemistry, Fudan University, Shanghai 200433, P. R. China*

‡*Hefei National Laboratory, Hefei 230088, P. R. China*

E-mail: xxchem@fudan.edu.cn; duansai@fudan.edu.cn

Contents

S1 Supplementary Tables **S3**

S1.1 Accuracy of selected level for energy refining S3

S1.2 Accuracy of various levels for evaluating the monohydration of $BN^{\bullet +}$ S3

S1.3 Reaction kinetics data of the rate-determining steps at $n = 2$ and 3 . . . . . S4

S2 Supplementary Figures **S5**

S2.1 Graphical representation of π electron distribution in BN and $BN^{\bullet +}$ S5

S2.2 IR spectrum of $BN^{\bullet +}-H_2O$ predicted at various levels S5

S2.3 Isomerization pathway between P_2^4 and P_3^4 . . . . S8

S2.4 Validation of the identification of Species 1-$H^+(H_2O)_{n-1}$ as the dominant ambient form of $BN^{\bullet +}-(H_2O)_n$ ($n \geq 2$) S9

References **S11**

S1 Supplementary Tables

S1.1 Accuracy of selected level for energy refining

Table S1: Relative free energies of different $BN^{\bullet+}-H_2O$ configurations optimized at the XYGJ-OS/may-cc-pVTZ[1–9] level with electronic energies at the DLPNO-CCSD(T)/aug-cc-pVTZ level[5–7,10–18] or the CCSD(T)/may-cc-pVTZ level[5–8,17–21] (unit: kJ/mol)

Configuration	ΔG (DLPNO-CCSD(T))	ΔG (CCSD(T))
m/p	0	0
o	0.39	0.35
C_1	0.67	0.37
o/m	0.71	0.50
C_4	1.42	1.75

S1.2 Accuracy of various levels for evaluating the monohydration of $BN^{\bullet+}$

Table S2: Thermodynamics of the monohydration of $BN^{\bullet+}$ at various levels

Level	$\Delta H^{\ominus}$ (kcal/mol)	$\Delta S^{\ominus}$ (cal/mol/K)
Experimental[22]	-8.8 ± 1.0	-18.9 ± 2.5
XYGJ-OS/may-cc-pVTZ[a 1–9]	-8.82	-21.76
XYGJ-OS/G3large[a 1–4,9,23–27]	-8.81	-21.70
RI-revDSD-PBEP86-D4/def2-TZVPPD[a 14,17,18,28–37]	-8.75	-21.27
RI-revDSD-PBEP86-D4/def2-QZVPPD[a 14,17,18,28–37]	-8.91	-21.05
RI-revDSD-PBEP86-D4/may-cc-pVTZ[a 5–8,14–18,28,29,32,33,35,36]	-8.82	-20.76
RI-revDSD-PBEP86-D4/aug-cc-pVTZ[a 5–7,14–18,28,29,32,33,35,36]	-8.80	-21.73
RI-ωB97X-2-D3(BJ)/def2-TZVPPD[a 14,17,18,30–41]	-8.76	-21.70
RI-ωB97X-2-D3(BJ)/def2-QZVPPD[a 14,17,18,30–41]	-8.85	-21.61
RI-ωB97X-2-D3(BJ)/may-cc-pVTZ[a 5–8,14–18,32,33,35,36,38–41]	-8.81	-21.35
RI-ωB97X-2-D3(BJ)/aug-cc-pVTZ[a 5–7,14–18,32,33,35,36,38–41]	-8.88	-21.68
RI-ωB97M-V/def2-TZVPPD[a 17,18,30–36,42–45]	-9.63	-22.73
XYGJ-OS/may-cc-pVTZ[b 1–9]	-8.81	-21.73
RI-ωB97X-D4/def2-TZVPPD[b 17,18,28,30–36,46,47]	-8.84	-20.99

[a] the electronic energies were refined at the CCSD(T)/may-cc-pVTZ level;[5–8,17–21]
[b] the electronic energies were refined at the DLPNO-CCSD(T) (tightPNO)/aug-cc-pVTZ level.[5–7,10–18]

S1.3 Reaction kinetics data of the rate-determining steps at $n = 2$ and 3

Table S3: Reaction kinetics data of the rate-determining steps at $n = 2$

T (K)	κ[48]	$\Delta G^{\ddagger}$ (kJ/mol)	$t_{1/2}$[49] (ms)
100	9.65×10^5	130.3	4.03×10^{52}
200	3.23	131.4	1.07×10^{24}
300	1.58	132.6	8.47×10^{12}
400	1.29	133.6	1.82×10^7
500	1.17	134.6	6.55×10^3
600	1.12	135.5	30.74
700	1.09	136.3	0.64

Table S4: Reaction kinetics data of the rate-determining steps at $n = 3$

T (K)	κ[48]	$\Delta G^{\ddagger}$ (kJ/mol)	$t_{1/2}$[49] (ms)
100	9.12×10^{13}	129.9	1.28×10^{44}
200	1.03×10^3	129.1	4.10×10^{20}
300	9.16	127.8	1.07×10^{11}
400	3.00	126.2	4.19×10^5
500	1.96	124.5	1.70×10^2
600	1.58	122.6	0.83
700	1.40	120.7	0.02

S2 Supplementary Figures

S2.1 Graphical representation of π electron distribution in BN and $BN^{\bullet+}$

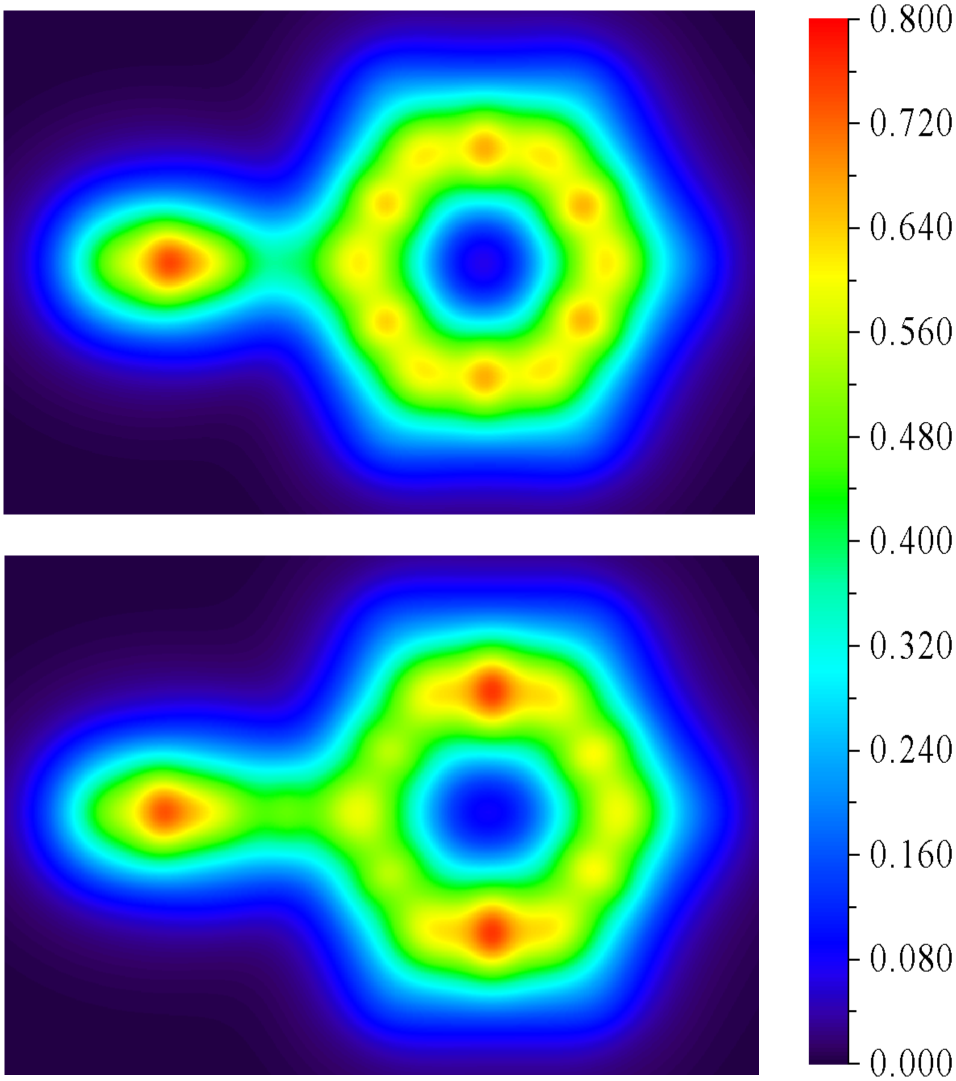

Figure S1: **LOL-π heat map[50–52] of BN (top) and $BN^{\bullet+}$ (bottom)**. The plotting plane is set parallel to the ring plane at 1.2 Bohr above for both molecules.

S2.2 IR spectrum of $BN^{\bullet+}-H_2O$ predicted at various levels

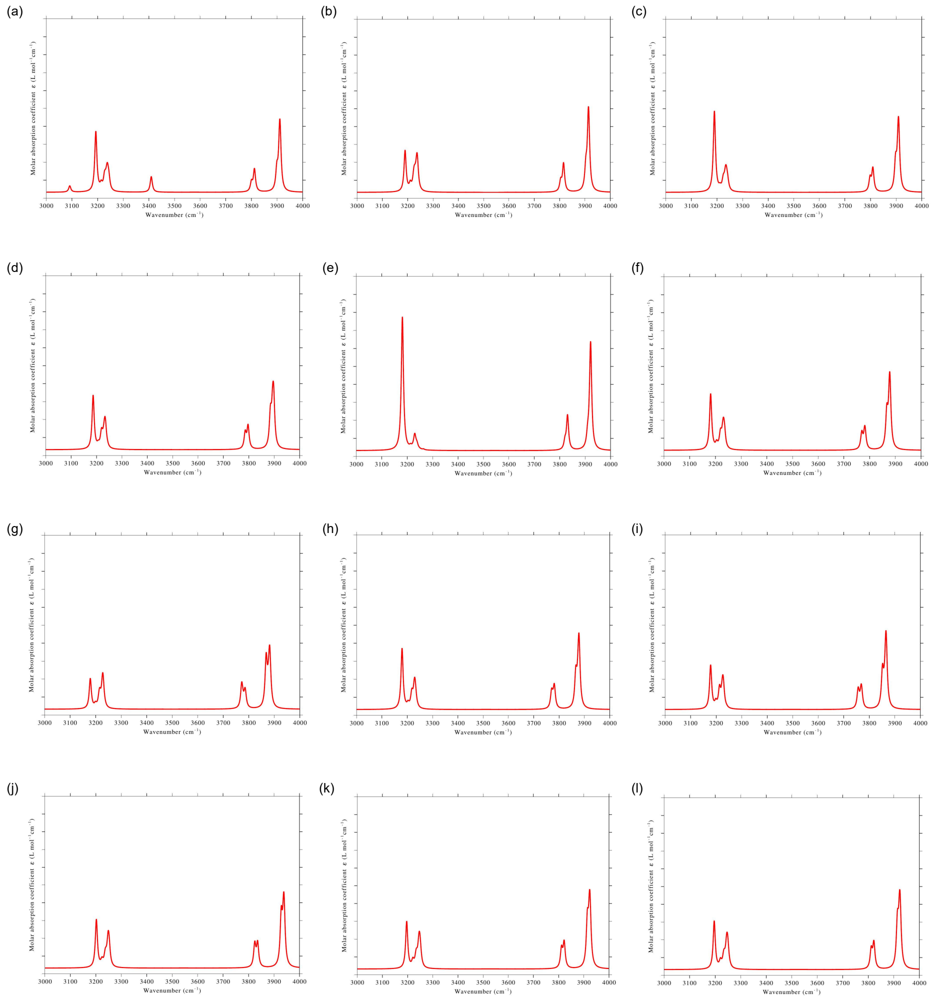

Figure S2: **Configuration-averaged IR spectrum of $BN^{\bullet+}-H_2O$ at the (a) RI-revDSD-PBEP86-D4/def2-TZVPPD; (b) RI-revDSD-PBEP86-D4/def2-QZVPPD; (c) RI-revDSD-PBEP86-D4/may-cc-pVTZ; (d) RI-revDSD-PBEP86-D4/aug-cc-pVTZ; (e) RI-ωB97M-V/def2-TZVPPD; (f) RI-ωB97X-2-D3(BJ)/def2-TZVPPD; (g) RI-ωB97X-2-D3(BJ)/def2-QZVPPD; (h) RI-ωB97X-2-D3(BJ)/may-cc-pVTZ; (i) RI-ωB97X-2-D3(BJ)/aug-cc-pVTZ; (j) XYGJ-OS/G3large; (k) XYGJ-OS/may-cc-pVTZ; (l) DLPNO-CCSD(T)(tightPNO)/aug-cc-pVTZ//XYGJ-OS/may-cc-pVTZ level.** Unless otherwise stated, all electronic energies were refined at the CCSD(T)/may-cc-pVTZ level.

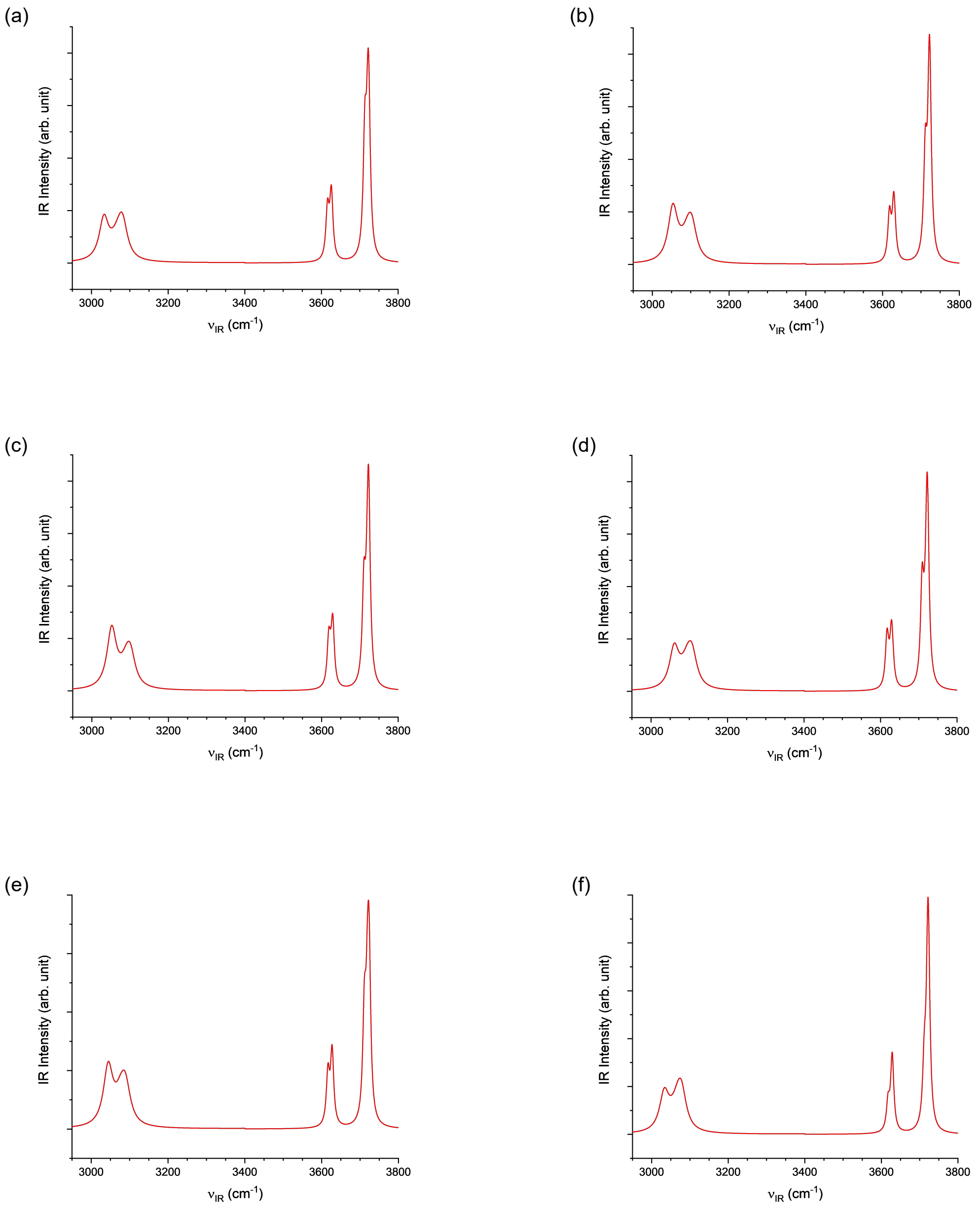

Figure S3: **Scaled configuration-averaged IR spectrum of $BN^{\bullet+}-H_2O$ at the (a) XYGJ-OS/may-cc-pVTZ; (b) RI-ωB97X-2-D3(BJ)/def2-TZVPPD; (c) RI-ωB97X-2-D3(BJ)/may-cc-pVTZ; (d) RI-ωB97X-2-D3(BJ)/aug-cc-pVTZ; (e) RI-revDSD-PBEP86-D4/aug-cc-pVTZ; (f) RI-revDSD-PBEP86-D4/def2-QZVPPD level.**

S2.3 Isomerization pathway between P_2^4 and P_3^4

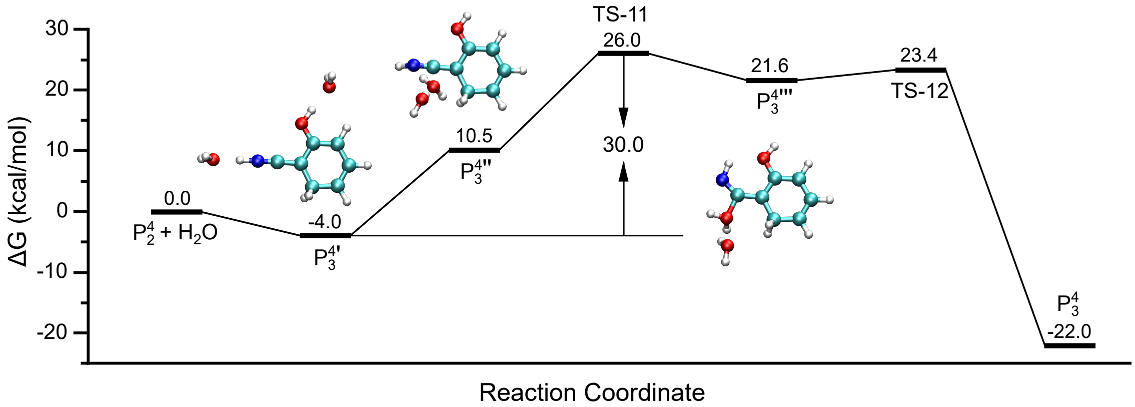

Figure S4: **Reaction pathway from $\mathbf{P_2^4}$ to $\mathbf{P_3^4}$ in the presence of free water.** Structures of local minima along the pathways are depicted, with barriers of the rate-determining steps labeled. "TS" represents transition state.

S2.4 Validation of the identification of Species 1-$H^+(H_2O)_{n-1}$ as the dominant ambient form of $BN^{\bullet+}-(H_2O)_n$ ($n \geq 2$)

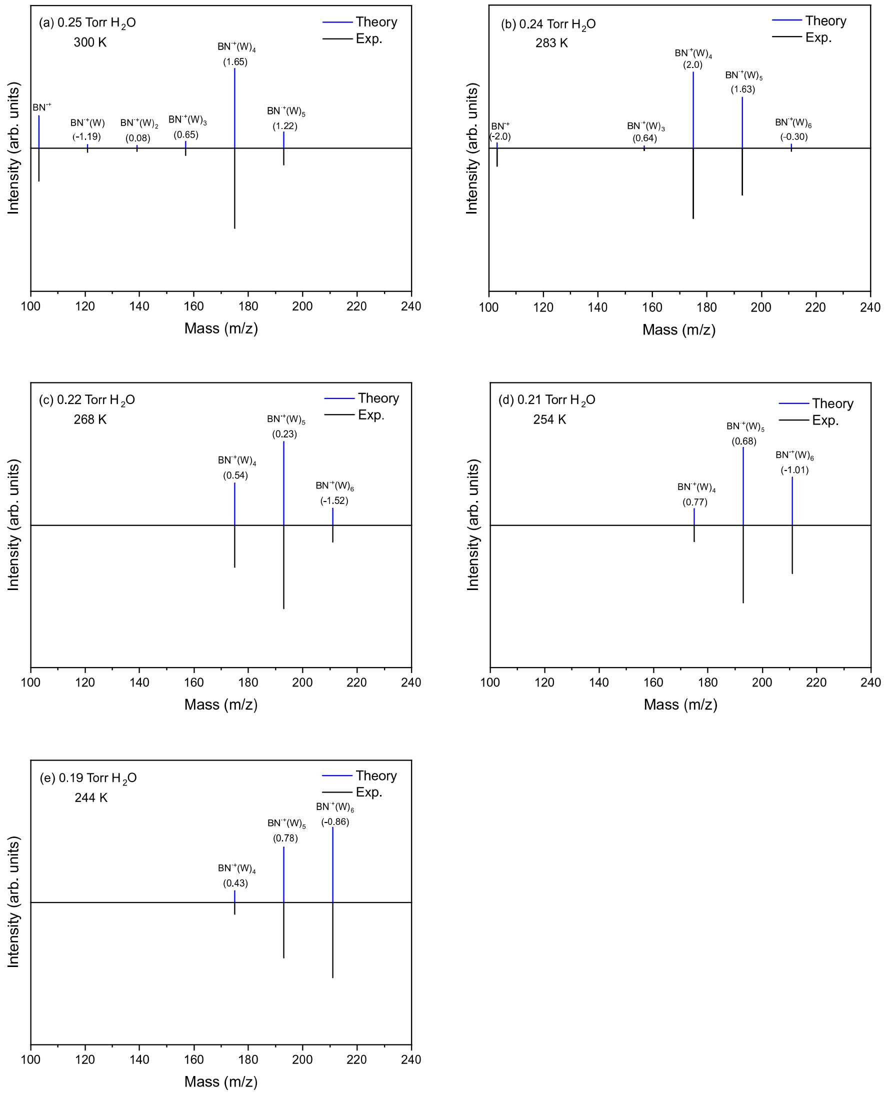

Figure S5: **Relative abundances of $BN^{\bullet+}-(H_2O)_n$ ($n \leq 6$) clusters at equilibrium under various conditions, where theoretical abundances were based on artificially corrected free energies and experimental data were measured by MSIM mass spectrum.**[22] Water vapor pressures and temperatures are indicated, with cluster sizes and corresponding free energy corrections labeled accordingly.

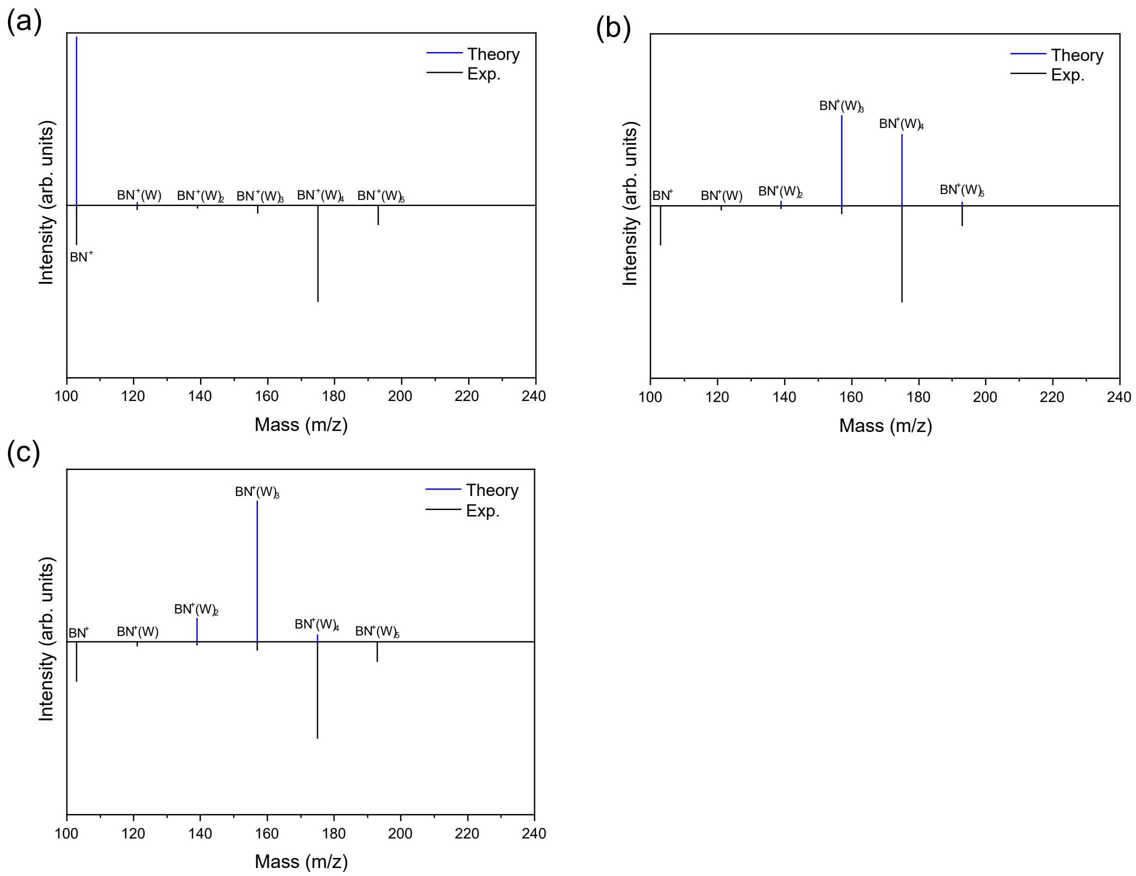

Figure S6: **Relative equilibrium abundances of $BN^{\bullet+}-(H_2O)_n$ ($n \leq 6$) clusters at 300 K, 0.25 Torr H_2O by taking (a) species from Ref. 22; (b) Species 3-$(H_2O)_{n-2}$; (c) Species 4-$(H_2O)_{n-2}$ as the dominant form, where experimental data were measured by MSIM mass spectrum.**[22] Cluster sizes were labeled accordingly.

References

(1) Zhang, I. Y.; Xu, X.; Jung, Y.; III, W. A. G. A fast doubly hybrid density functional method close to chemical accuracy using a local opposite spin ansatz. *Proceedings of the National Academy of Sciences* **2011**, *108*, 7650–7655.

(2) Su, N. Q.; Zhang, I. Y.; Xu, X. Analytic derivatives for the XYG3 type of doubly hybrid density functionals: Theory, implementation, and assessment. *Journal of Computational Chemistry* **2013**, *34*, 1759–1774.

(3) Su, N. Q.; Xu, X. Beyond energies: geometry predictions with the XYG3 type of doubly hybrid density functionals. *Chem. Commun.* **2016**, *52*, 13840–13860.

(4) Gu, Y.; Zhu, Z.; Xu, X. Second-Order Analytic Derivatives for XYG3 Type of Doubly Hybrid Density Functionals: Theory, Implementation, and Application to Harmonic and Anharmonic Vibrational Frequency Calculations. *Journal of Chemical Theory and Computation* **2021**, *17*, 4860–4871.

(5) Dunning, T. H. Gaussian basis sets for use in correlated molecular calculations. I. The atoms boron through neon and hydrogen. *The Journal of Chemical Physics* **1989**, *90*, 1007–1023.

(6) Davidson, E. R. Comment on "Comment on Dunning's correlation-consistent basis sets". *Chemical Physics Letters* **1996**, *260*, 514–518.

(7) Kendall, R. A.; Dunning, J., Thom H.; Harrison, R. J. Electron affinities of the first-row atoms revisited. Systematic basis sets and wave functions. *The Journal of Chemical Physics* **1992**, *96*, 6796–6806.

(8) Papajak, E.; Zheng, J.; Xu, X.; Leverentz, H. R.; Truhlar, D. G. Perspectives on basis sets beautiful: Seasonal plantings of diffuse basis functions. *Journal of Chemical Theory and Computation* **2011**, *7*, 3027–3034.

(9) Frisch, M. J. et al. Gaussian 09, Revision D.01. 2013; `https://gaussian.com`.

(10) Riplinger, C.; Neese, F. Natural triple excitations in local coupled cluster calculations with pair natural orbitals. *The Journal of Chemical Physics* **2013**, *138*, 134101.

(11) Guo, Y.; Zahariev, F. N.; Neese, F. Communication: An improved linear scaling perturbative triples correction for DLPNO-CCSD(T). *The Journal of Chemical Physics* **2018**, *148*, 011101.

(12) Guo, Y.; Riplinger, C.; Liakos, D. G.; Becker, U.; Saitow, M.; Neese, F. Linear scaling perturbative triples correction approximations for open-shell domain-based local pair natural orbital coupled cluster singles and doubles theory [DLPNO-CCSD(T0/T)]. *The Journal of Chemical Physics* **2020**, *152*, 024116.

(13) Weigend, F. A fully direct RI-HF algorithm: Implementation, optimized auxiliary basis sets, demonstration of accuracy and efficiency. *Physical Chemistry Chemical Physics* **2002**, *4*, 4285–4291.

(14) Weigend, F.; Häser, M.; Patzelt, H.; Ahlrichs, R. RI-MP2: Optimized auxiliary basis sets and demonstration of efficiency. *Chemical Physics Letters* **1998**, *294*, 143–152.

(15) Weigend, F. Hartree–Fock exchange fitting basis sets for H to Rn. *Journal of Computational Chemistry* **2008**, *29*, 167–175.

(16) Weigend, F.; Köhn, A.; Hättig, C. Efficient use of the correlation consistent basis sets in resolution of the identity MP2 calculations. *The Journal of Chemical Physics* **2002**, *116*, 3175–3183.

(17) Neese, F.; Wennmohs, F.; Becker, U.; Riplinger, C. The ORCA quantum chemistry program package. *The Journal of Chemical Physics* **2020**, *152*, 224108.

(18) Neese, F. Software update: The ORCA program system—Version 5.0. *WIREs Computational Molecular Science* **2022**, *12*, e1606.

(19) Purvis, G. D.; Bartlett, R. J. A full coupled-cluster singles and doubles model: The inclusion of disconnected triples. *The Journal of Chemical Physics* **1982**, *76*, 1910–1918.

(20) Raghavachari, K.; Trucks, G. W.; Pople, J. A.; Head-Gordon, M. A fifth-order perturbation comparison of electron correlation theories. *Chemical Physics Letters* **1989**, *157*, 479–483.

(21) Watts, J. D.; Gauss, J.; Bartlett, R. J. Coupled-cluster methods with noniterative triple excitations for restricted open-shell Hartree–Fock and other general single determinant reference functions. Energies and analytical gradients. *The Journal of Chemical Physics* **1993**, *98*, 8718–8733.

(22) Mason, K. A.; Pearcy, A. C.; Christensen, Z. A.; Attah, I. K.; Meot-Ner (Mautner), M.; El-Shall, M. S. Water-Assisted Proton Transfer in the Sequential Hydration of Benzonitrile Radical Cation $C_6H_5CN^{\bullet +}-(H_2O)_n$: Transition to Hydrated Distonic Cation $^{\bullet}C_6H_4CNH^+(H_2O)$n with n $\geq$ 4. *Journal of the American Chemical Society* **2022**, *144*, 9684–9694.

(23) Ditchfield, R.; Hehre, W. J.; Pople, J. A. Self-consistent molecular-orbital methods. IX. An extended Gaussian-type basis for molecular-orbital studies of organic molecules. *The Journal of Chemical Physics* **1971**, *54*, 724–728.

(24) Krishnan, R.; Binkley, J. S.; Seeger, R.; Pople, J. A. Self-consistent molecular orbital methods. XX. A basis set for correlated wave functions. *The Journal of Chemical Physics* **1980**, *72*, 650–654.

(25) Clark, T.; Chandrasekhar, J.; Spitznagel, G. W.; von Ragué Schleyer, P. Efficient diffuse function-augmented basis sets for anion calculations. III. The 3-21+G basis set for first-row elements, Li–F. *Journal of Computational Chemistry* **1983**, *4*, 294–301.

(26) Frisch, M. J.; Pople, J. A.; Binkley, J. S. Self-consistent molecular orbital methods

25. Supplementary functions for Gaussian basis sets. *The Journal of Chemical Physics* **1984**, *80*, 3265–3269.

(27) Curtiss, L. A.; Raghavachari, K.; Redfern, P. C.; Rassolov, V.; Pople, J. A. Gaussian-3 (G3) theory for molecules containing first and second-row atoms. *The Journal of Chemical Physics* **1998**, *109*, 7764–7776.

(28) Caldeweyher, E.; Bannwarth, C.; Grimme, S. Extension of the D3 dispersion coefficient model. *The Journal of Chemical Physics* **2017**, *147*, 034112.

(29) Santra, G.; Sylvetsky, N.; Martin, J. M. L. Minimally empirical double-hybrid functionals trained against the GMTKN55 database: revDSD-PBEP86-D4, revDOD-PBE-D4, and DOD-SCAN-D4. *The Journal of Physical Chemistry A* **2019**, *123*, 5129–5143.

(30) Weigend, F.; Ahlrichs, R. Balanced basis sets of split valence, triple zeta valence and quadruple zeta valence quality for H to Rn: Design and assessment of accuracy. *Physical Chemistry Chemical Physics* **2005**, *7*, 3297–3305.

(31) Rappoport, D.; Furche, F. Property-optimized Gaussian basis sets for molecular response calculations. *Journal of Chemical Physics* **2010**, *133*, 134105.

(32) Eichkorn, K.; Treutler, O.; Ohm, H.; Häser, M.; Ahlrichs, R. Auxiliary basis sets to approximate Coulomb potentials. *Chemical Physics Letters* **1995**, *240*, 283–290.

(33) Neese, F. An improvement of the resolution of the identity approximation for the formation of the Coulomb matrix. *Journal of Computational Chemistry* **2003**, *24*, 1740–1747.

(34) Weigend, F. Accurate Coulomb-fitting basis sets for H to Rn. *Physical Chemistry Chemical Physics* **2006**, *8*, 1057–1065.

(35) Neese, F.; Wennmohs, F.; Hansen, A.; Becker, U. Efficient, approximate and parallel Hartree–Fock and hybrid DFT calculations. A 'chain-of-spheres' algorithm for the Hartree–Fock exchange. *Chemical Physics* **2009**, *356*, 98–109.

(36) Helmich-Paris, F.; Neese, F. An improved chain of spheres exchange algorithm. *The Journal of Chemical Physics* **2021**, *155*, 104109.

(37) Hellweg, A.; Rappoport, D. Development of new auxiliary basis functions of the Karlsruhe segmented contracted basis sets including diffuse basis functions (def2-SVPD, def2-TZVPPD, and def2-QVPPD) for RI-MP2 and RI-CC calculations. *Physical Chemistry Chemical Physics* **2007**, *9*, 563–571.

(38) Chai, J.-D.; Head-Gordon, M. Long-range corrected double-hybrid density functionals. *The Journal of Chemical Physics* **2009**, *131*, 174105.

(39) Grimme, S.; Antony, J.; Ehrlich, S.; Krieg, H. A consistent and accurate ab initio parametrization of density functional dispersion correction (DFT-D) for the 94 elements H-Pu. *The Journal of Chemical Physics* **2010**, *132*, 154104.

(40) Grimme, S.; Ehrlich, S.; Goerigk, L. Effect of the damping function in dispersion corrected density functional theory. *Journal of Computational Chemistry* **2011**, *32*, 1456–1465.

(41) Mehta, N.; Goerigk, L. Semi-empirical or non-empirical double-hybrid density functionals: which are more robust? *Physical Chemistry Chemical Physics* **2018**, *20*, 23158–23174.

(42) Vydrov, O. A.; Voorhis, T. V. Nonlocal van der Waals density functional: The simpler the better. *The Journal of Chemical Physics* **2010**, *133*, 244103.

(43) Hujo, W.; Grimme, S. Performance of the van der Waals density functional VV10 and (hybrid) GGA variants for thermochemistry and noncovalent interactions. *Journal of Chemical Theory and Computation* **2011**, *7*, 3866–3871.

(44) Mardirossian, N.; Head-Gordon, M. ωB97M-V: A combinatorially optimized, range-

separated hybrid, meta-GGA density functional with VV10 nonlocal correlation. *The Journal of Chemical Physics* **2016**, *144*, 214110.

(45) Lehtola, S.; Steigemann, C.; Oliveira, M. J. T.; Marques, M. A. L. Recent developments in libxc — A comprehensive library of functionals for density functional theory. *SoftwareX* **2018**, *7*, 1–5.

(46) Mardirossian, N.; Head-Gordon, M. ωB97X-V: A 10-parameter, range-separated hybrid, generalized gradient approximation density functional with nonlocal correlation, designed by a survival-of-the-fittest strategy. *Physical Chemistry Chemical Physics* **2014**, *16*, 9904–9924.

(47) Najibi, A.; Goerigk, L. DFT-D4 counterparts of leading meta-GGA and hybrid density functionals for energetics and geometries. *Journal of Computational Chemistry* **2020**, *41*, 2562–2572.

(48) Eckart, C. The Penetration of a Potential Barrier by Electrons. *Phys. Rev.* **1930**, *35*, 1303–1309.

(49) Eyring, H. The Activated Complex and the Absolute Rate of Chemical Reactions. *Chemical Reviews* **1935**, *17*, 65–77.

(50) Schmider, H.; Becke, A. Chemical content of the kinetic energy density. *Journal of Molecular Structure: THEOCHEM* **2000**, *527*, 51–61.

(51) Lu, T.; Chen, F. Multiwfn: A multifunctional wavefunction analyzer. *Journal of Computational Chemistry* **2012**, *33*, 580–592.

(52) Lu, T. A comprehensive electron wavefunction analysis toolbox for chemists, Multiwfn. *The Journal of Chemical Physics* **2024**, *161*, 082503.
